# Case Report: Long-term survival of adenoid cystic carcinoma with central airway stenosis treated by bronchoscopic ablation combined with para-toluenesulfonamide injection

**DOI:** 10.3389/fonc.2026.1772650

**Published:** 2026-03-17

**Authors:** Yedan Wu, Li Zhang

**Affiliations:** Department of Respiratory & Critical Care Medicine, Tianjin Chest Hospital, Tianjin, China

**Keywords:** airway obstruction, brachytherapy, debulking, electrocautery, laser, para-toluenesulfonamide, tracheal adenoid cystic carcinoma

## Abstract

**Patient concerns:**

A 70-year-old man with a primary tracheal adenoid cystic carcinoma (TACC) located at 5 cm of the tracheal process.

**Interventions:**

We adopted bronchoscopic ablation technology, including cryotherapy of probe tumor reduction and thermal ablation, which lasted for 7 years. After that, flexible mechanical tumor reduction was used to remove the tumor, and para-toluenesulfonamide (PTS) was injected to treat any residual tumors. During the treatment, we found that scarring-induced stenosis of the trachea near the superior aspect of the tumor, under these circumstances, we performed bronchoscopic laser treatment to remove scar tissue and relieve central airway obstruction.

**Outcomes:**

The combined use of bronchoscopic laser treatment and PTS injection represents a promising treatment, especially suitable for patients who are not suitable for surgical removal. This case highlights the importance of individualized treatment strategies in managing complex airway obstruction, improving the quality of life of patients, and providing symptom relief for patients with advanced tumors.

## Introduction

Tracheal tumors are relatively rare among primary respiratory malignant tumors, and most of them are malignant tumors, adenoid cystic carcinoma (ACC) accounts for about 10-20% of these cases (1–5). The clinical manifestations of primary tracheal adenoid cystic carcinoma (TACC) usually include symptoms such as dyspnea, cough, shortness of breath, hemoptysis, wheezing, chest pain, inspiratory wheezing, neck swelling and hoarseness. In this case report, we introduced a patient whose TACC is located at 5 cm of the tracheal process. Due to the patient’s age, surgical removal is not an option. Therefore, we adopted bronchoscopic ablation technology, including cryotherapy of probe tumor reduction and thermal ablation, which lasted for seven years. After that, flexible mechanical tumor reduction was used to remove the tumor, and para-toluenesulfonamide (PTS) was injected to treat any residual tumors.

## Case report

A 70-year-old man with a 40-year history of smoking was hospitalized for persistent coughing and gradually worsening motorized dyspnea. The patient has no history of other chronic diseases or tuberculosis. In May 2017, chest CT scans found a 2-cm-wide tumor in the trachea, which almost blocked the airway.

**Figure 1:**
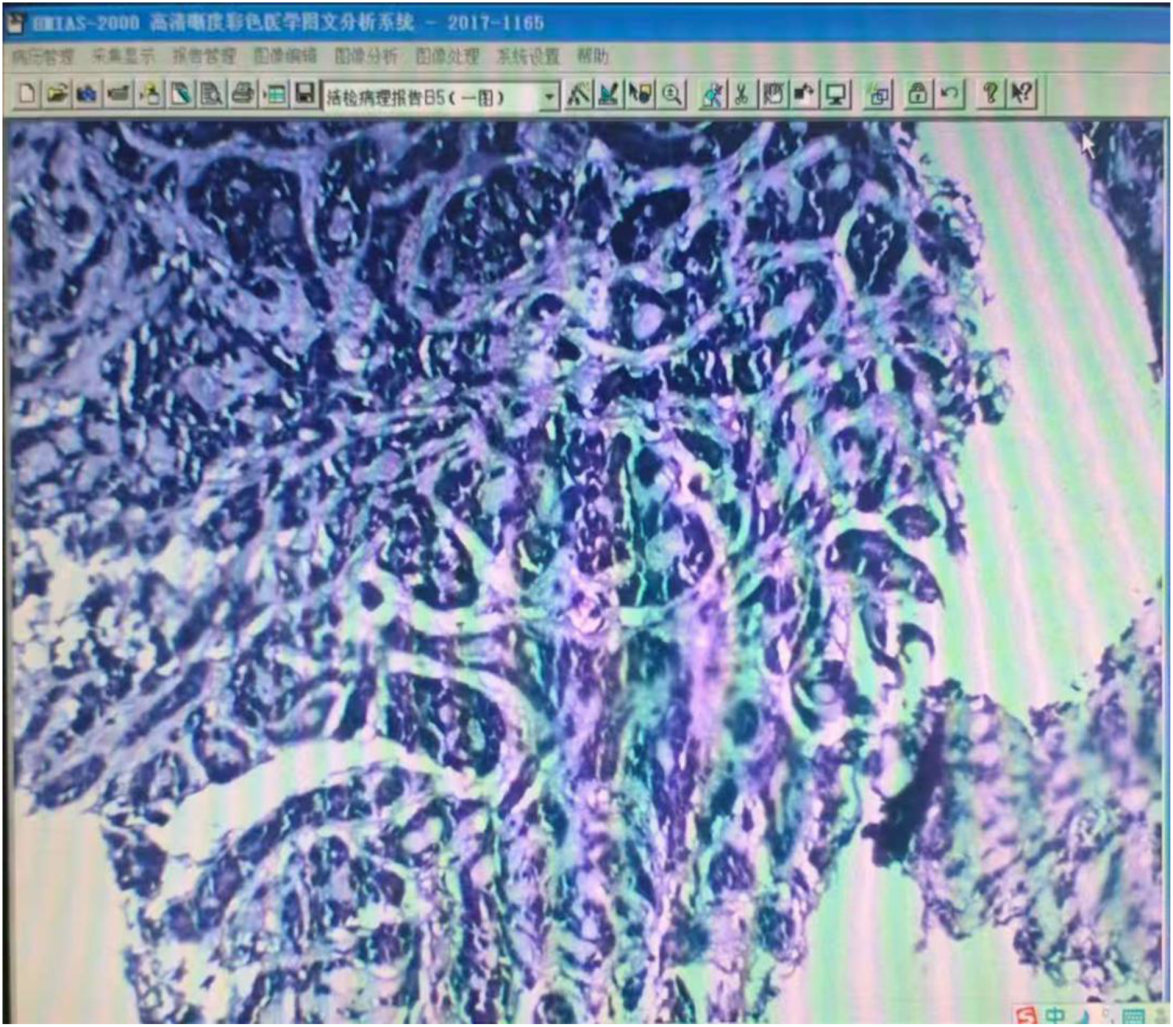
Pathology image.

**Figure 2:**
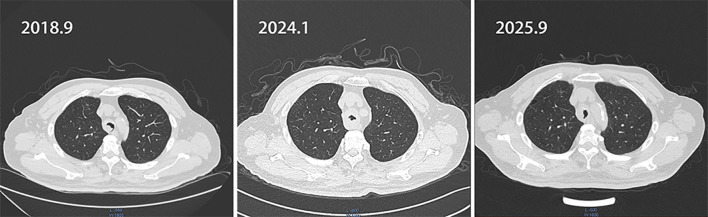
CT indicates recurrent tracheal tumor leading to tracheal stenosis.

**Figure 3:**
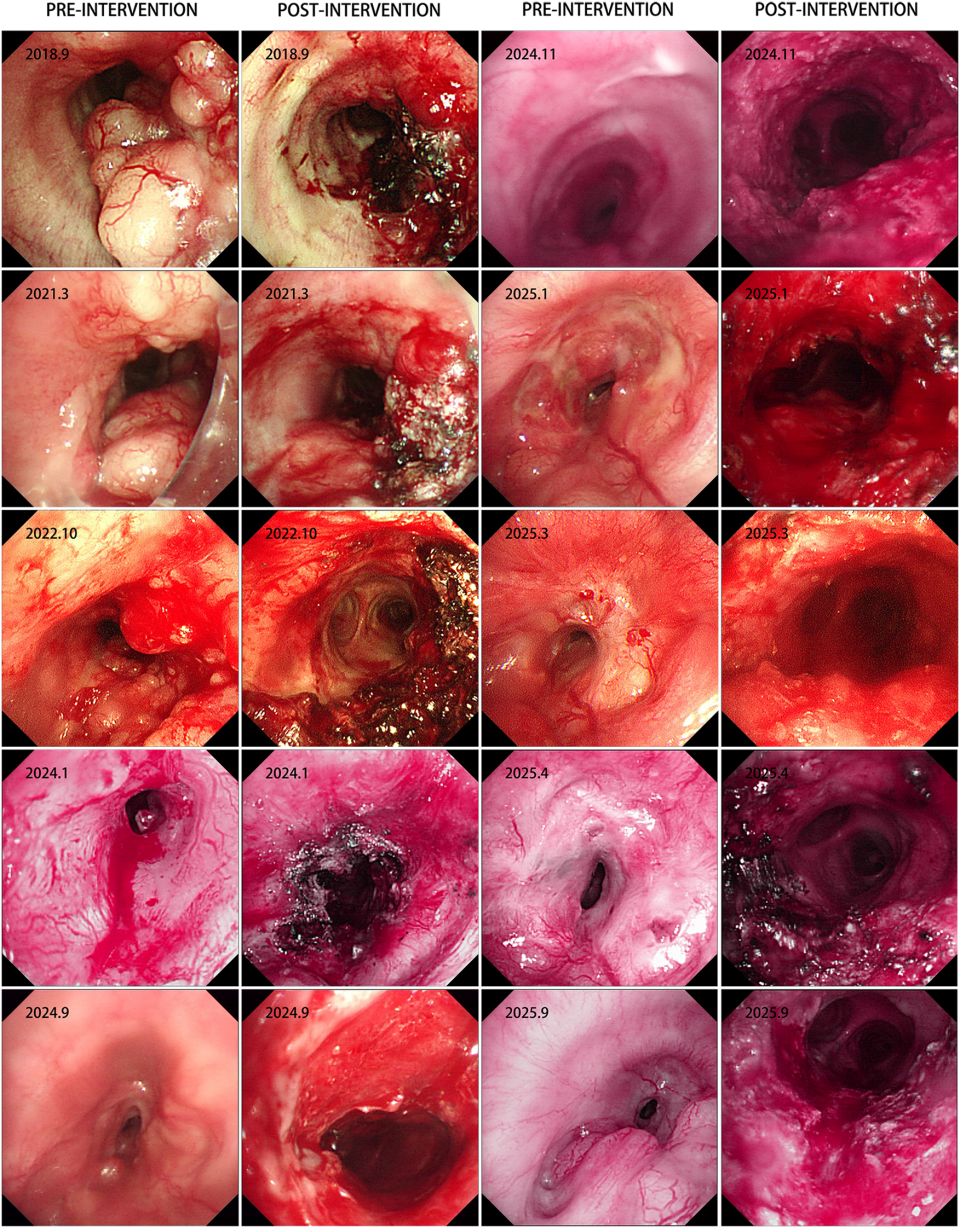
Interventional therapy under bronchoscopy.

Pathological examination of the submitted specimen (main bronchus biopsy) revealed malignant tumor tissue (**Figure 1**). Based on the cytomorphologic features and the immunohistochemical staining profile, the findings were consistent with adenoid cystic carcinoma. Immunohistochemistry showed: TTF-1 (1+), S-100 (focally positive), p63 (positive), CEA (negative), Ki-67 (positive; >1%), p53 (positive), CD56 (negative), and synaptophysin (Syn) (negative). In order to alleviate the symptoms and reduce the risk of suffocation, we performed intrabronchial electrocautery, cryotherapy and argon plasma coagulation(APC) to reduce the tumor with the probe. In the next several years (2017-2024), the patient had right lung metastasis, followed by double lung metastasis. Despite these conditions, the patient refused any anti-tumor treatment. Various bronchoscopic interventions are carried out every year, including cryotherapy probe reduction, electrocautery, biopsy forceps and lasso to manage and control the tumor. Unfortunately, in January 2024, the tumor growth accelerated and the central airway stenosis gradually worsened. Flexible mechanical tumor reduction combined with PTS intratumor injection was adopted. However, the patient experienced repeated central airway stenosis. In April 2025, we encountered a new issue. Bronchoscopy revealed scarring-induced stenosis of the trachea near the superior aspect of the tumor, under these circumstances, we performed bronchoscopic laser treatment to remove scar tissue and relieve central airway obstruction. The corresponding CT changes, bronchoscopy images, and details of the intervention are depicted in **Figures 2**, **3**, and presented in **Table 1**, respectively.

**Table 1:** Types of interventional therapy.

	Electrocautery	APC	Cryotherapy	Laser therapy	PTS
2017.5	**√**	**√**	**√**		
2018.9	**√**		**√**		
2019.11	**√**		**√**		
2021.3	**√**	**√**	**√**		
2022.10	**√**		**√**		
2024.1	**√**	**√**	**√**		**√**
2024.5	**√**	**√**	**√**		**√**
2024.9	**√**				
2024.11	**√**	**√**	**√**		**√**
2025.1	**√**		**√**		**√**
2025.3	**√**	**√**	**√**		**√**
2025.4	**√**		**√**	**√**	
2025.6			**√**	**√**	
2025.9	**√**	**√**	**√**		**√**

√ indicates operations that have been implemented, while blank spaces indicate operations that have not been performed.

Technical details: Cryotherapy was performed using a COOLAND K320 system (COOLAND) with a flexible respiratory endoscopy cryoprobe (KRT181050). Depending on the lesion characteristics and procedural goal, either a freeze–cut or freeze–thaw technique was applied. For freeze–cut debulking, freezing was typically maintained for 5–15 s followed by thawing for 10–15 s. For freeze–thaw ablation, freezing was maintained for 60–90 s, followed by thawing for 10–15 s. Electrocautery/APC was delivered using an ERBE VIO200S electrosurgical unit coupled with APC2 (ERBE Elektromedizin, Tübingen, Germany). An argon applicator (ERBE 20132-221) and a needle electrode (ALTON AF-1810DZ) were used as appropriate. The cutting power was set to 50–65 W. APC settings were 30–50 W with an argon gas flow of 1–2 L/min. To reduce the risk of airway fire during APC, the inspiratory oxygen fraction (FiO_2_) was maintained at <40% throughout energy delivery. Contact laser therapy was performed with the JXmedex LaserPro980 system (JXmedex), using a wavelength of 980 nm. A laser fiber (FEF2.2-SMA) with a tip (MTRG3.5) was applied in single-pulse mode at 10–15 W, according to the extent of residual tumor and airway patency. Following mechanical debulking, intratumoral PTS injection was performed under direct bronchoscopic visualization under EBUS guidance. For each session, a commercially available PTS kit (5 mL/1.65 g PTS + 2 mL diluent) was reconstituted immediately before administration per the manufacturer’s instructions. A 21-gauge mucosal injection needle was used to deliver multipoint injections (approximately 3–5 sites) into the tumor base and residual tumor tissue. At each site, 0.1–0.3 mL was injected while gradually withdrawing the needle to facilitate intratumoral dispersion. After injection, the airway was routinely observed for acute bleeding or other abnormalities, and the bronchial lumen was irrigated with normal saline as needed. Regarding depth control, the injection depth was controlled by real-time EBUS visualization in combination with the predetermined needle extension of the bronchoscopic injection needle (and by monitoring tactile resistance during needle advancement). The needle was advanced until the tip was visualized within the tumor on EBUS, and the injection was performed only after this intratumoral position was confirmed.

In the course of treatment, the improvement in the patient’s quality of life was most notable in the following aspects:

Symptom Relief: After bronchoscopic ablation and laser therapy, the patient experienced significant relief from dyspnea (mMRC score decreased from grade 3 to grade 1), as well as improvements in coughing and wheezing. Following restoration of central airway patency, the patient’s exercise tolerance improved, allowing the patient to engage in daily activities without the need for long-term home oxygen therapy.

**Figure 4:**
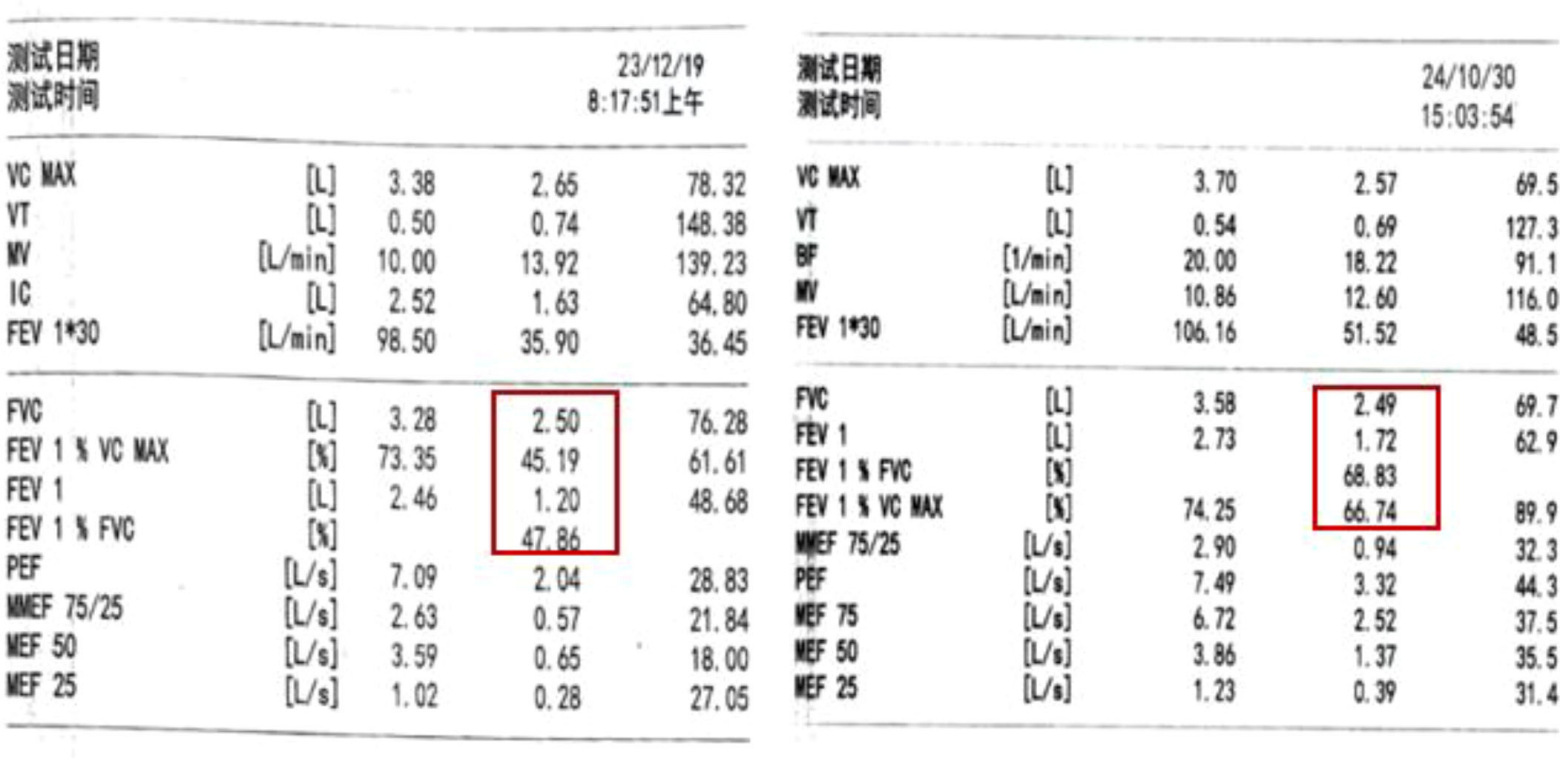
Pulmonary function comparison.

Quality of Life Assessment Tools: This study is a retrospective case report, and thus, standardized quality of life instruments (such as EORTC QLQ-C30 or SF-36) were not used for systematic assessment. Instead, we indirectly assessed improvements in quality of life through clinical follow-up records, symptom scores (mMRC), frequency of re-interventions, and subjective feedback from the patient. For example, the frequency of bronchoscopic interventions decreased from 3–4 times per year to 1–2 times post-treatment, and there was a significant reduction in emergency visits. Compared to before treatment, lung function has significantly improved. Compared to 2023, FEV1 and FEV1/FVC in 2024 have significantly increased, as shown in **Figure 4**.

Long-term Maintenance of Quality of Life: Despite the presence of bilateral lung metastases, the patient was able to maintain relatively stable airway patency and symptom control through regular localized interventions, thus avoiding the need for tracheostomy or long-term mechanical ventilation. The patient retained good self-care ability and psychological well-being.

In future studies, we plan to incorporate standardized quality of life assessment tools to more objectively measure the impact of interventional treatments on the patient’s overall quality of life.

## Discussion

This case highlights the complex management problems of TACC with central airway obstruction in a 70-year-old male patient. The patient’s long-term smoking history and repeated central airway stenosis after multiple bronchoscopic interventions have brought significant clinical challenges.

TACC is a rare malignancy characterized by slow growth, a tendency for perineural and local invasion, and a high risk of late distant metastasis. Surgical resection remains the first-line treatment for localized disease and offers favorable long-term survival outcomes when complete resection is achievable. In an institutional series of 38 patients with tracheal ACC, definitive surgical resection (with or without adjuvant radiotherapy) was associated with excellent 5-year overall survival and freedom from local progression (6). In contrast, radiotherapy alone provided reasonable local control in patients with unresectable or inoperable cases, emphasizing the importance of a multidisciplinary approach to managing this rare tumor type. However, in the current case, surgical resection was not selected due to several patient-specific factors. At 70 years of age, with a long history of smoking and bilateral lung metastases, the patient’s overall surgical risk was high. The patient explicitly refused both surgery and systemic chemotherapy, and the tumor’s location in the middle trachea required extensive resection, further complicating the decision. The anticipated risk of postoperative complications, such as anastomotic leaks and restenosis, was substantial. The patient’s subjective refusal, combined with the tumor’s extent (approximately 6 cm in the trachea), made surgical resection and anastomosis technically challenging and suboptimal in this scenario.

For patients who are not candidates for surgery due to comorbidities, tumor extent, or refusal of resection, bronchoscopic ablative interventions can offer effective palliation and symptom relief. Techniques such as laser therapy, electrocautery, cryotherapy, and other thermal ablation modalities have been demonstrated to safely relieve central airway obstruction, improve symptoms, and facilitate subsequent definitive therapy (7). However, despite their benefits, recurrence and restenosis remain common challenges. In this case, the obstruction was caused by an endoluminal tumor, and although stenting is commonly used for malignant central airway obstruction, it was not chosen as a treatment option. Stent placement would not reduce the tumor volume and could exacerbate local irritation, promote granulation tissue formation, and be ineffective in controlling tumor growth. Additionally, the patient had already undergone multiple bronchoscopic interventions, and the insertion of a stent would have carried increased risks, including infection, displacement, and mucus plugging.

Given the persistence of residual tumor tissue after multiple ablation treatments, PTS injection was selected as a supplementary local therapy to delay tumor growth and complement the ablation efforts. PTS, a low-molecular-weight antitumor agent, has shown promising antineoplastic activity in preclinical models, demonstrating significant tumor growth inhibition and histological necrosis with minimal adjacent tissue injury when compared to traditional ethanol injection (8). These findings suggest the potential utility of PTS as a local therapeutic adjunct. Although clinical data on PTS in human tracheal or airway tumors remain limited, early clinical trials and animal studies support further investigation into its safety and efficacy when administered intratumorally (9). This approach also avoids the systemic side effects associated with chemotherapy. The simplicity of the injection procedure, its repeatability, and its ability to provide localized tumor control make it an ideal option for this patient, aligning with the goal of maintaining airway patency and controlling local tumor progression.

The overarching principle involved an individualized, stepwise treatment strategy, dynamically tailored according to the obstruction mechanism (tumor versus scar), patient tolerance, and observed local efficacy.

To address different stages and mechanisms of obstruction(**Table 2**): early tumors were debulked with cryo-/thermo-ablative techniques; larger masses were rapidly opened with mechanical debulking; scar stenosis was targeted with laser therapy; and residual tumor was controlled with adjunctive PTS injection. The initial treatments consisted of combination therapy, including cryotherapy and electrocautery. At that time, PTS had not yet been approved in our country, and no published reports were available regarding its therapeutic effects in ACC. With respect to laser therapy, first, the cost of the equipment is prohibitively high. Second, for the removal of tumor masses, laser therapy does not demonstrate any particularly clear advantages over high-frequency electrosurgery or cryotherapy. The subsequent introduction of PTS injection and laser therapy was based on two considerations. First, literature evidence supports the efficacy of PTS injection in the treatment of ACC. Second, some patients developed scar hyperplasia following local excision. To mitigate local scar formation, we selected an upgraded laser-based approach.

**Table 2:** Indications for escalation or modification of therapy.

Time period	Interventions	Primary indications and rationale
2018–2023	Bronchoscopic cryotherapy, electrocautery, APC	New or recurrent endobronchial tumors; minimally invasive debulking and rapid airway recanalization; suitable for patients ineligible for surgery
January 2024	Mechanical debulking + intratumoral PTS injection	Accelerated tumor growth with significant luminal narrowing; rapid mechanical reduction combined with local drug therapy for residual lesions
2024–2025	Repeated mechanical debulking + PTS injection	Tumor regrowth and recurrent symptoms; maintaining local control and delaying restenosis
April 2025	Bronchoscopic laser therapy	Scar stenosis becoming the main cause of obstruction; precise laser resection of scar tissue to restore airway anatomy

Because the base of ACC is often very broad, complete resection is extremely difficult. Therefore, preventing recurrence and avoiding rapid worsening of airway obstruction within a short period of time constitutes a particularly important therapeutic objective. Compared with mechanical debulking or thermal ablative tumor-reduction techniques, PTS is primarily administered by injection into the root of the resection site, with the aim of preventing tumor recurrence. Firstly, the rationale for selecting PTS over other intratumoral injection agents is that PTS is a lipid-soluble local chemotherapeutic drug capable of remaining within tumor tissue for a prolonged period and being released slowly, thereby enhancing local therapeutic effects (10). Compared with anhydrous ethanol, bleomycin, and related agents, PTS induces less damage to normal tissues (11) and is therefore more suitable for repeated administration. Preliminary basic research further indicates that PTS exhibits certain antitumor activity against adenoid cystic carcinoma (8), providing a theoretical basis for its use in the present case. Secondly, regarding the specific contribution of PTS compared with repeat ablation, ablation primarily addresses visible tumors within the lumen, whereas PTS can target sub-visible or deeply infiltrating components, thereby delaying tumor regeneration. The “supplemental injection” after ablation is intended to prolong the intervention-free interval and reduce the total number of treatments. Thirdly, effectiveness evaluation indicators include objective measures: bronchoscopic assessment of tumor volume (percentage of lumen obstruction), the time interval between two interventions, and changes in tumor cell activity on pathological biopsy, as well as symptom indicators, integrating changes in the mMRC dyspnea score and the cough VAS score.

In this case, the occurrence of airway scar stenosis may be related to the following mechanisms: Mechanisms supported by the literature (evidence): Repeated bronchoscopic interventions, particularly thermal ablation and/or mechanical manipulation, can cause local injury, which in turn triggers inflammation and, ultimately, fibrosis (12). Evidence suggests that direct tumor invasion of the airway wall can cause structural disruption, and scar formation may readily occur during subsequent airway wall repair and healing (13). Observations and hypotheses in the present case: 1. The stenosis developed after multiple ablation procedures, and the effectiveness of laser therapy for the scar supports the hypothesis that iatrogenic injury was the primary cause; 2. Anhydrous ethanol (used as the solvent for PTS) may exacerbate local inflammation; however, its independent effect remains unclear; 3.There are no direct reports indicating fibrogenic effects of PTS itself; nevertheless, further research is required to determine whether local reactions after injection contribute to stenosis. The cicatricial stenosis observed was likely attributable to prior mechanical debulking or thermal ablation procedures. During the patient’s earlier treatments, the extent of cryoablation, thermal ablation, or mechanical resection was smaller than that of the last few treatments. However, even so, we did not identify any cicatricial tissue formation causing airway stenosis in the airway. Instead, after the final injection of PTS, the patient developed localized cicatricial airway stenosis, which was also confirmed pathologically. Of course, adenoid cystic carcinoma was also identified in the pathological specimens, indicating recurrence of cancer tissue. Another possible explanation is that the disease itself (TACC) can lead to tumor-related remodeling of the airway wall, resulting in stenosis even after tumor control.

ACC is characterized by slow growth and delayed metastasis, and its natural disease course often spans several years or even more than a decade. In this case, a survival time of >7 years partly reflects the indolent biological behavior of this disease. However, the subsequent development of metastases in the right lung and then bilaterally indicates that the disease had progressed to an advanced stage. Under these circumstances, ongoing bronchoscopic interventions played a pivotal role in maintaining airway patency and preventing asphyxia and acute respiratory failure, thereby enabling the patient to preserve a relatively good quality of life and functional status even without receiving systemic therapy. Compared with the outcomes reported in the literature for unresectable TACC (median survival approximately 5–8 years, often relying on surgery and/or radiotherapy), this case suggests that, for patients who are unable to undergo curative treatment, proactive local airway management may serve as an important strategy for prolonging survival. Although adenoid cystic carcinoma is a malignancy that grows very slowly and metastasizes very late, the lesion in this patient was located in the central airway and caused prominent symptoms of airway obstruction, even leading to episodes of asphyxia. Therefore, without airway intervention, the patient’s survival would certainly have been adversely affected. Notably, before the most recent several treatments, the patient’s dyspnea had already become very severe; if the tumor had been allowed to continue growing, the patient would likely have developed asphyxia with a life-threatening risk.

This study is a single-center retrospective case report and has several limitations. First, the sample size is limited to a single case, and extrapolation of the conclusions should be approached with caution. Second, treatment decisions were based on individualized clinical judgment, and no control group was available. Third, quality-of-life assessment was not performed using standardized instruments. Fourth, the causal relationship between PTS injection and cicatricial stenosis remains speculative. Therefore, we do not recommend considering bronchoscopic laser therapy combined with PTS injection as a standard treatment strategy; rather, we emphasize that it provides an individualized, multimodal interventional approach for patients with complex central airway obstruction who are not candidates for surgery. Future prospective studies are needed to validate the safety and long-term efficacy of this combined strategy.

To date, there are no large-scale clinical studies reporting outcomes of combined bronchoscopic ablation and local PTS injection specifically for tracheal ACC. However, our case contributes to the emerging evidence suggesting that the integration of mechanical airway debulking with localized pharmacologic tumor suppression can maintain airway patency while exerting local tumor control. Such individualized multimodal strategies may expand therapeutic options for patients who are medically inoperable and warrant further investigation in prospective studies.

## Conclusion

In summary, the occurrence of airway scar stenosis is mainly related to tissue damage, inflammatory reaction and airway wall healing process, especially when it involves repeated intervention and local treatment. Although the main function of PTS is to inhibit tumor growth and reduce residual tumor tissue, its potential side effects, especially local inflammation and fibrosis, may increase the risk of airway scar stenosis. During the treatment process, especially in cases involving airway tumours, these potential side effects must be closely monitored and appropriate interventions must be taken to reduce the risk of airway stenosis.

## Data Availability

The original contributions presented in the study are included in the article/supplementary material. Further inquiries can be directed to the corresponding author.
